# MRPL42 is activated by YY1 to promote lung adenocarcinoma progression

**DOI:** 10.7150/jca.52277

**Published:** 2021-03-01

**Authors:** Wei Jiang, Chengpeng Zhang, Yunteng Kang, Xiaojun Yu, Pei Pang, Guangbin Li, Yu Feng

**Affiliations:** 1 Department of Thoracic Surgery, the First Affiliated Hospital of Soochow, University, Suzhou, Jiangsu, China.; 2 Department of Pathology, the First Affiliated Hospital of Soochow University, Suzhou, Jiangsu, China.

**Keywords:** MRPL42, YY1, proliferation, metastasis, transcriptional regulation

## Abstract

Mammalian mitochondrial ribosomal proteins are a group of protein factors encoded by nuclear genes, responsible for the synthesis of proteins in mitochondria. As a member of mitochondrial ribosomal proteins, MRPL42 (mitochondrial ribosomal protein L42) belongs to 28S and 39S subunits. The current literature showed that its role in lung adenocarcinoma (LUAD) was not clear. We found that MRPL42 was highly expressed in early-stage LUAD tissues and cell lines, and remarkably related to the prognosis of patients. Knockdown of MRPL42 could reduce the proliferation and colonization, promote cell cycle arrest in G1/S phase, and weaken the migration and invasion ability of LUAD cells* in vitro*. Moreover, depletion of MRPL42 also inhibited tumor growth *in vivo*. Bioinformatics analysis found that YY1 may bind to the promoter region upstream of the MRPL42 gene to promote the transcription of MRPL42, which was verified by the ChIP and Dual luciferase reporter assay. QRT-PCR confirmed that knocking down YY1 could attenuate the expression of MRPL42. In summary, MRPL42 acts as an oncogene in LUAD, and its expression level is regulated by YY1.

## Introduction

In various countries, lung cancer (LC) is recognized as one of the most malignant tumors (accounting for 11.6% of all cancer types), and it is also one of the leading causes of cancer deaths (accounting for 18.4% of all cancer deaths) 1-3. According to histological classification, LC is mainly divided into small cell lung cancer (SCLC) and non-small cell lung cancer (NSCLC) (about 85%), and lung adenocarcinoma (LUAD) is the most common histological subtype of NSCLC, accounting for more than 40% of LC 4. Clinically, with the development of imaging examinations, the disease spectrum of early-stage LC is mainly characterized by solitary lung nodules, ground glass nodules, and sub-centimeter nodules. Early surgical treatment often indicates a good prognosis. At present, video-assisted thoracoscopic surgical treatment is a main choice 5-7. In the past two years, robot-assisted pneumonectomy has gradually been carried out clinically 8. However, less than 40% of patients are still diagnosed when the lesion is confined to the lungs, which is still a problem for patients 9. For advanced LC, even though the related therapies have made great progress in recent years (including immunotherapy, targeted therapy), the 5-year overall survival rate of LUAD patients is still not high because of its high invasiveness and metastasis 10, 11. Therefore, a deeper understanding of the signaling pathways and molecular mechanisms may brew more treatment options for LUAD.

Mammalian mitochondrial ribosomal proteins are encoded by nuclear genes, responsible for protein synthesis in the mitochondria. Mitochondrial ribosomal protein L41 (MRPL41) could inhibit the growth of xenograft tumors by stabilizing P53 12. MRPL42 is found in 28S and 39S mitochondrial ribosome subunits. Knocking down (MRPL42) could inhibit glioma cell growth by inducing cell cycle arrest and apoptosis 13. MRPL42 could also be used to predict the progression of LC and the overall survival of patients 14. However, its molecular mechanism in LUAD is currently unclear.

In this study, based on the microarray data set of early-satge LUAD patients, we evaluated the expression of MRPL42. A series of *in vitro* and* in vivo* experiments were performed to confirm the mechanism through which that MRPL42 regulates the proliferation and metastasis of LC cells (A549 and H1299).

## Materials and Methods

### Microarray data processing and bioinformatics analysis

mRNA expression patterns of MRPL42 in normal tissue samples and early-stage LUAD tissue samples were obtained from the Gene Expression Omnibus (GEO) database (https://www.ncbi.nlm.nih.gov/geo) 15. GSE21933 16, GSE32863 17 and GSE33532 18 were selected for further study. The Cancer Genome Atlas (TCGA) database (https://tcga-data.nci.nih.gov) was based to analyze the mRNA expression of MRPL42 in normal and LUAD cancer tissues 19. Clinical Proteomic Tumor Analysis Consortium database (CPTAC) (https://proteomics.cancer.gov/data-portal) and The Human Protein Atlas database (https://www.proteinatlas.org/) were used to examine the protein expression of MRPL42 in LUAD tissue samples 20, 21. The YY1 binding motif in the promoter region of MRPL42 was predicted by JASPAR database (http://jaspar.genereg.net/) and PROMO database (http://alggen.lsi.upc.es/cgibin/promo_v3/promo/promoinit.cgi?dirDB=TF_8.3) 22, 23.

### Human LUAD samples

56 pairs of LUAD tissues and adjacent tissues were collected from patients undergoing thoracic surgery in the First Affiliated Hospital of Soochow University from January 2018 to January 2019. The patients were randomly selected, all showing primary LUAD and, no use of radiotherapy or chemotherapy before surgery. This study was approved by the Research Ethics Committee of the First Affiliated Hospital of Soochow University, and written informed consents were obtained from all patients. The tissues were immediately placed in liquid nitrogen after surgical removal.

### Cell culture

All cell lines (16HBE, A549, SPCA1, and H1299) were purchased from the Chinese Academy of Sciences Cell Bank (Shanghai, China) and cultured in DMEM medium (Gibco, NY, USA) supplemented with 10% fetal bovine serum (Gibco), 100 U/mL penicillin (Gibco) and 100 μg/mL streptomycin (Gibco) at 37 °C in humidified air containing 5% CO_2_.

### Quantitative real-time PCR (qRT-PCR)

According to the manufacturer's protocol, total RNA was extracted from tissues and cells, and cDNA was synthesized using PrimeScript RT kit (Takara, Shiga Prefecture, Japan). SYBR Green Mix II (Takara) was chosen to quantify mRNA expression on CFX Connect fluorescent quantitative PCR system (Bio-Rad, California, USA). The primers were synthesized by Genechem (Shanghai, China). The primer sequence is as follows: MRPL42 forward: 5'-CTGACTTCTGATGGCAGGAC-3', reverse: 5'-TCCATGAGGATACCAACGGT-3'; YY1 forward: 5'-AAGAGCGGCAAGAAGAGTTAC-3', reverse: 5'- CAACCACTGTCTCATGGTCAATA-3'; GAPDH forward: 5'-TGACTTCAACAGCGACACCCA-3', reverse: 5'-CACCCTGTTGCTGTAGCCAAA-3'. Denaturation was performed at 95 °C for 30 seconds, followed by 40 cycles at 92 °C s for 5 seconds, at 55 °C for 30 seconds, and then at 72 °C for 30 seconds.

### Cell transfection

LV-sh-ctrl, LV-sh-MRPL42, si-ctrl and si-YY1 were designed and synthesized by Genechem (Shanghai, China). Cells transfected with the lentiviruses were screened with puromycin to obtain stably transfected cell lines. siRNA transfection was performed using Lipofectamine 3000 (Invitrogen, California, USA), according to the manufacturer's protocol.

### Western blot analysis

The protein was extracted with a whole cell protein extraction kit (NCM Biotech, Suzhou, China) and Phosphatase Inhibitor Cocktail (NCM Biotech), and the concentration was determined using BCA analysis (NCM Biotech) according to the manufacturer's instructions. A total of 30 µg of protein was subjected to 10% or 12% PAGE (EpiZyme, Shanghai, China), and then transferred to a 0.22 µm PVDF (EpiZyme) membrane. Having been incubated with anti-MRPL42, anti-p-Akt, anti-Vimentin, anti-GAPDH and anti-YY1 at 4 °C overnight, the protein content was detected by the Enhanced Chemiluminescent reagent (NCM Biotech) on the next day.

### Cell proliferation assays

For colony formation assay, stably transfected cells were seeded into six-well plates at a density of 1×10^3^ cells per well. After 14 days, the colonies were stained with crystal violet staining solution (Beyotime, Shanghai, China). Then, the number of colonies was manually counted. For CCK-8 assay, 100 μL of cell suspension containing 1×10^3^ cells was added into each well of the 96-well plate. Then, the absorbance at 450 nm was measured at 24, 48, 72 and 96 h after transfection, respectively, using the CCK-8 solution (10 μl) (Dojindo, Kumamoto, Japan). For cell cycle analysis, after adding the propidium iodide (PI) (Vazyme, Nanjing, China), the number of positive cells was analyzed using FACScan. The proportion of cells in the G1, S, and G2/M phases were counted and compared.

### Cell migration and invasion assays

For migration assays, 2×10^4^ stably transfected cells in serum-free DMEM medium were placed into the upper chamber of the insert (8-μm pore size; Millipore, Massachusetts, USA). For invasion assays, 1×10^5^ cells in serum-free DMEM medium were seeded into the upper chamber of an insert coated with Matrigel (Invitrogen). DMEM medium containing 10% FBS was placed into the lower chamber. After an incubation of 36 h, the upper layer of cells was wiped with a cotton swap, and the cells on the lower surface were fixed in methanol and, stained with crystal violet staining solution (Beyotime), and photographed using a microscope.

### Animal experiments

Six nude mice (4-5 weeks old) were purchased from the Animal Experiment Center of Soochow University. All animal experiments were conducted under the supervision of the Animal Protection and Utilization Committee of Soochow University. sh-MRPL42 cells and sh-ctrl cells were injected into the left and right armpit, respectively. Twenty-four days after injection, all nude mice were sacrificed, photographed and tumor nodules were weighed.

### Chromatin immunoprecipitation (ChIP)

ChIP analysis was performed using Magna ChIP Chromatin Immunoprecipitation Kit (Millipore). The chromatin was cross-linked with formaldehyde, sonicated into small fragments, and immunoprecipitated with anti-YY1 or anti-IgG antibodies bound to magnetic beads. After decrosslinking, the enrichment of specific fragments was determined by qRT-PCR.

### Dual luciferase reporter assay

For promoter analysis, the wild-type (wt) or mutant-type (mt) sequence of the MRPL42 promoter region in which the presumed YY1-binding site was 5′- CAAGATGGCGGG-3′ was constructed using the pGL3 vector (Promega, Wisconsin, USA). Wt or mt plasmid was co-transfected with si-YY1 or si-Ctrl using Lipofectamine™ 3000 (Invitrogen). After 48 h, the luciferase activities were detected using dual luciferase reporter assays (Promega).

### Immunohistochemical (IHC) analysis

All nude mouse tumors were fixed in 4% formalin and embedded in paraffin. After random sectioning, the endogenous peroxide and protein were blocked, and the sections were incubated with diluted specific anti-Ki-67 or anti-vimentin at 4 °C overnight. The next day, the sections were incubated with the secondary antibody at 37 °C for 1 hour. The slides were stained with 3,3-diaminobenzidine solution for 3 minutes and counterstained with hematoxylin. Two pathologists examined tumor sections to determine the percentage of positive tumors and the intensity of cell staining.

### Statistical analysis

Data from at least three independent replicates were shown as Mean ± SD. SPSS software, 24.0 (SPSS Inc., Chicago, IL, USA) and Graphpad Prism 7.0 (San Diego, CA, USA) were used for one-way ANOVA among multiple groups and two-tailed Student t-test between two groups. Differences were considered as statistically significant if P<0.05.

## Results

### MRPL42 was overexpressed in LUAD tissues and cell lines

**Figure 1:**
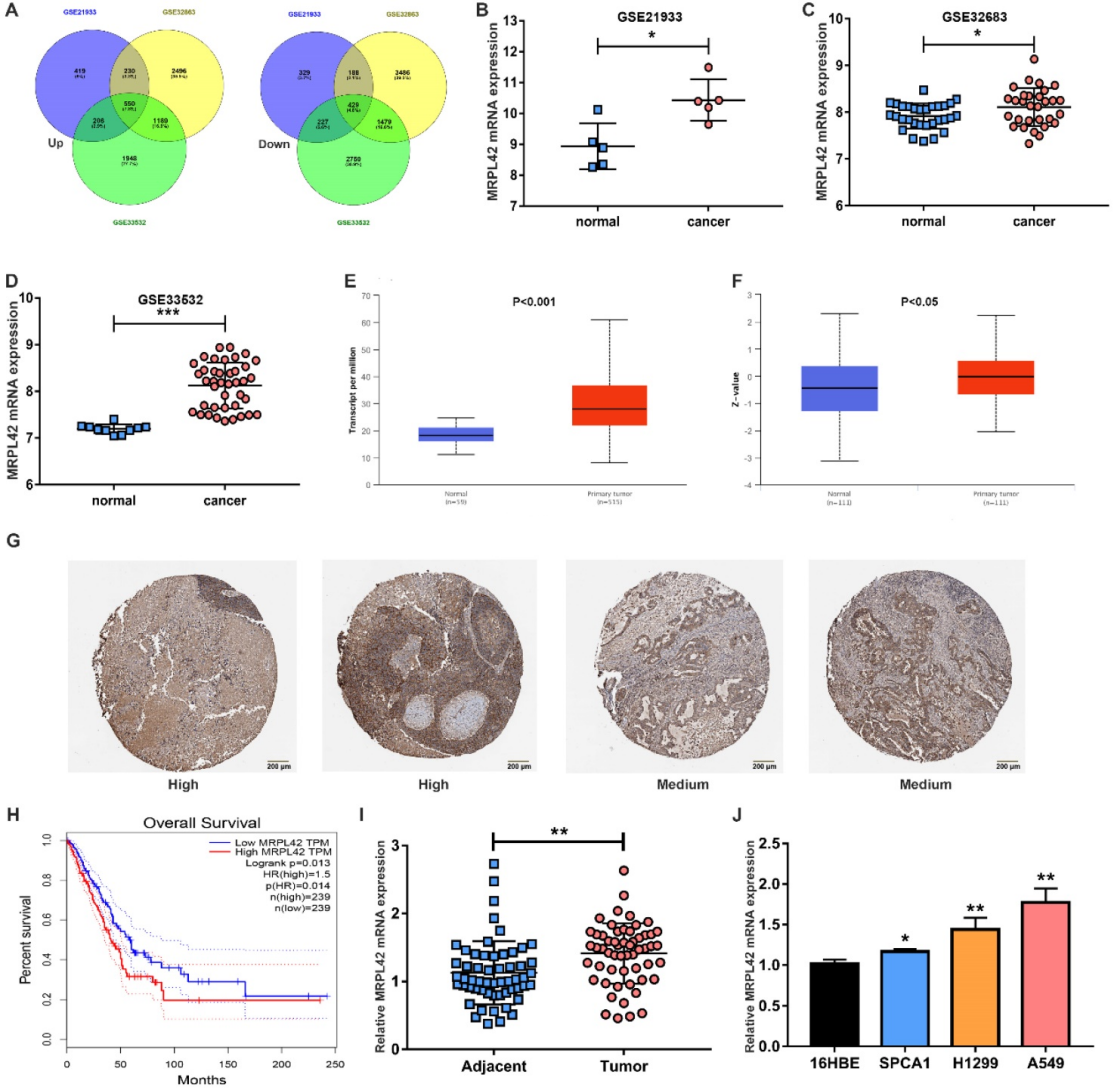
**MRPL42 was upregulated in LUAD tissues and cells.** (A) Dysregulated genes in GSE21933, GSE32863 and GSE33532. (B) The MRPL42 mRNA expression level reported in the GSE21933. (C) The MRPL42 mRNA expression level reported in the GSE32863. (D) The MRPL42 mRNA expression level reported in the GSE33532. (E) The MRPL42 mRNA expression level reported in the TCGA database. (F) The MRPL42 protein expression level reported in the CPTAC database. (G) MRPL42 protein expression in LUAD tissues. (H) The relationship between the expression level of MRPL42 and the prognosis of patients. (I) The MRPL42 mRNA expression in LUAD patients was detected by qRT-PCR. (J) The MRPL42 mRNA expression in LUAD cell lines and 16HBE. Data are represented as mean ± SD. (*P<0.05; **P<0.01; ***P<0.001).

In order to find genes that are dysregulated in the development of LUAD, especially in early-stage LUAD (stage I and II), 4 microarray samples (GSE21933, GSE32863 and GSE33532) were selected. As indicated in previous literature, we selected MRPL42 out of 550 up-regulated genes for further analysis (Figure 1A). As shown in Figure 1B, 1C and 1D, the mRNA expression of MRPL42 in tumor tissues (n=5, 30, 40, respectively) of patients with early-stage LUAD was significantly higher than that in normal tissues (n=5, 30, 40, respectively). Similarly, in the TCGA database, compared with normal samples (n=59), the expression of mRNA MRPL42 in tumor tissue samples (n=515) was significantly increased (Figure 1E). In the CPTAC database, the expression of MRPL42 protein in tumor tissues (n=111) was significantly higher than that in normal tissues (n=111) (Figure 1F). In the Human Protein Atlas database, MRPL42 was moderately or highly expressed in tumor tissues (Figure 1G). Further analysis found that the expression of MRPL42 was also related to the patient's prognosis. The prognosis of LUAD patients with high expression of MRPL42 was poor (Figure 1H). The expression of MRPL42 was also increased abnormally in the tissues of LUAD patients (n=56) undergoing surgery at our institution (Figure 1I). The expression level of MRPL42 was related with the patient's tumor size and lymph node metastasis, but not with the patient's gender, age, and smoking (Table 1). Similarly, as indicated in Figure 1J, the expression of MRPL42 in LUAD cell lines (A549, SPCA1 and H1299) was significantly higher than that in normal bronchial epithelium (16HBE). Taking together, MRPL42 was abnormally highly expressed in tumor tissues, indicating its oncogenic role in the occurrence and development of LUAD.

### MRPL42 downregulation inhibited LUAD cell proliferation *in vitro*

**Figure 2:**
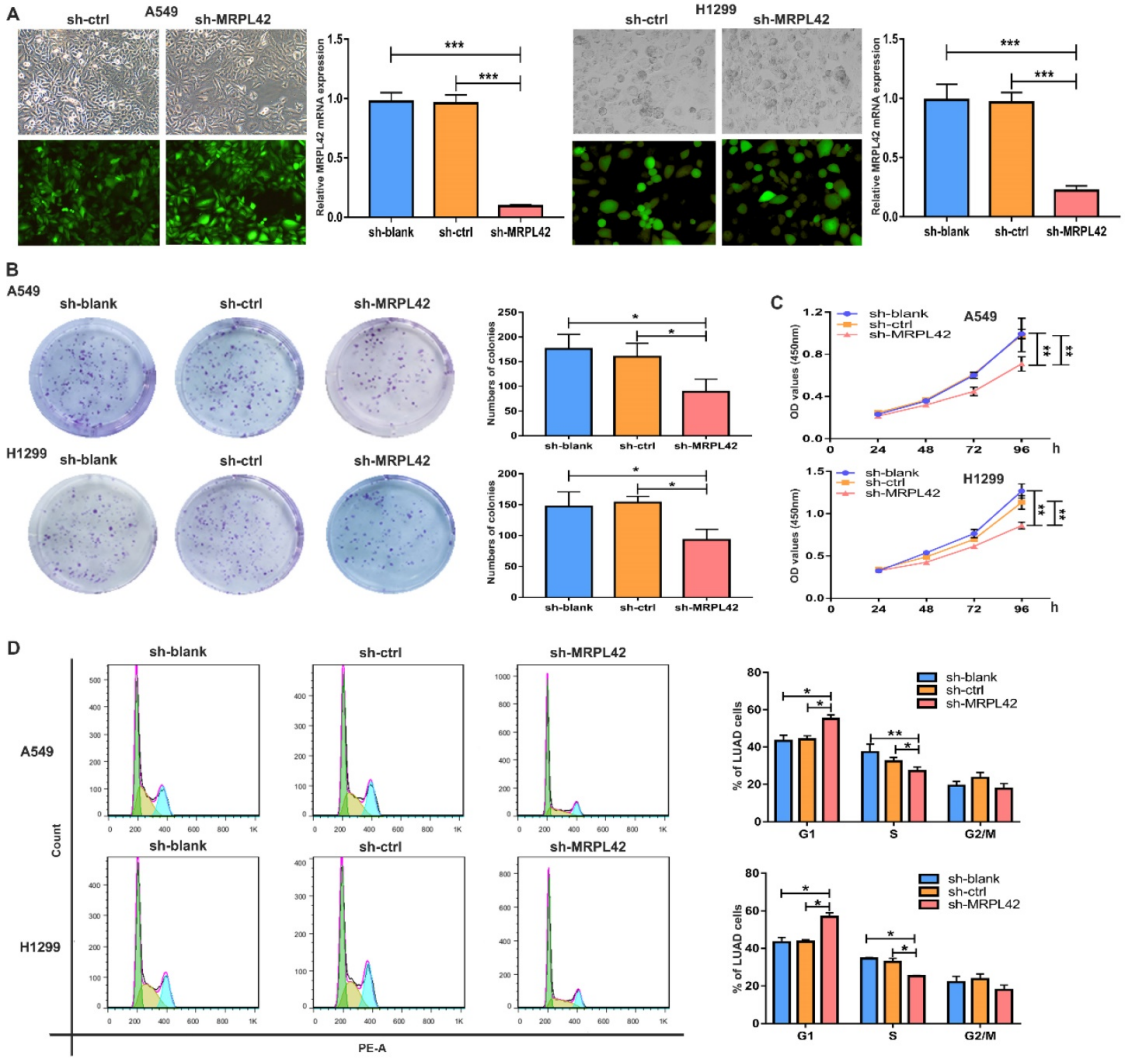
** MRPL42 knockdown inhibited LUAD cell proliferation.** (A) A549 cells and H1299 cells were transfected with sh-blank, sh-ctrl or sh-MRPL42 and detected by qRT-PCR. (B) Colony forming assay comparing the sh-blank, sh-ctrl, sh-MRPL42 groups. (C) CCK-8 assay comparing the sh-blank, sh-ctrl, sh-MRPL42 groups. (D) The effect of sh-blank, sh-ctrl and sh-MRPL42 on cell cycle distribution of NSCLC cells. Data are represented as mean ± SD. (*P<0.05; **P<0.01; ***P<0.001).

To further explore the biological functions of the abnormally highly expressed MRPL42, we conducted a series of *in vitro* experiments. A549 and H1299 were selected for lentiviral transfection to reduce the expression level of MRPL42. The transfected cells were respectively named sh-blank, sh-ctrl and sh-MRPL42. After stable expression, fluorescence microscope, qRT-PCR and Western blot were performed to detect the transfection efficiency. As shown in Figure 2A and Figure S1, the expression of MRPL42 in A549 and H1299 cells in sh-MRPL42 group was significantly was suppressed, compared with that in sh-blank group and the sh-ctrl group. In the colony formation experiment, the colony-forming ability of both A549 cells or H1299 cells in sh-MRPL42 group was significantly weakened (Figure 2B). In CCK-8 assay, the growth rate of the cells in sh-MRPL42 group began to slow down at three days after transfection (Figure 2C). To determine whether MRPL42 has an effect on cell cycle, we examined the loss-of-MRPL42 cell lines using flow cytometry. As shown in Figure 2D, the G1 and S phases of cell cycle in sh-MRPL42 group cells was significantly shorten. In addition, the expression of p-Akt in sh-MRPL42 group was also significantly lower than that in sh-blank group and sh-ctrl group (Figure S1). Taken together, MRPL42 downregulation inhibited the proliferation of LUAD cells through inducing G1/S arrest.

### MRPL42 knockdown suppressed cell migration and invasion* in vivo*

**Figure 3:**
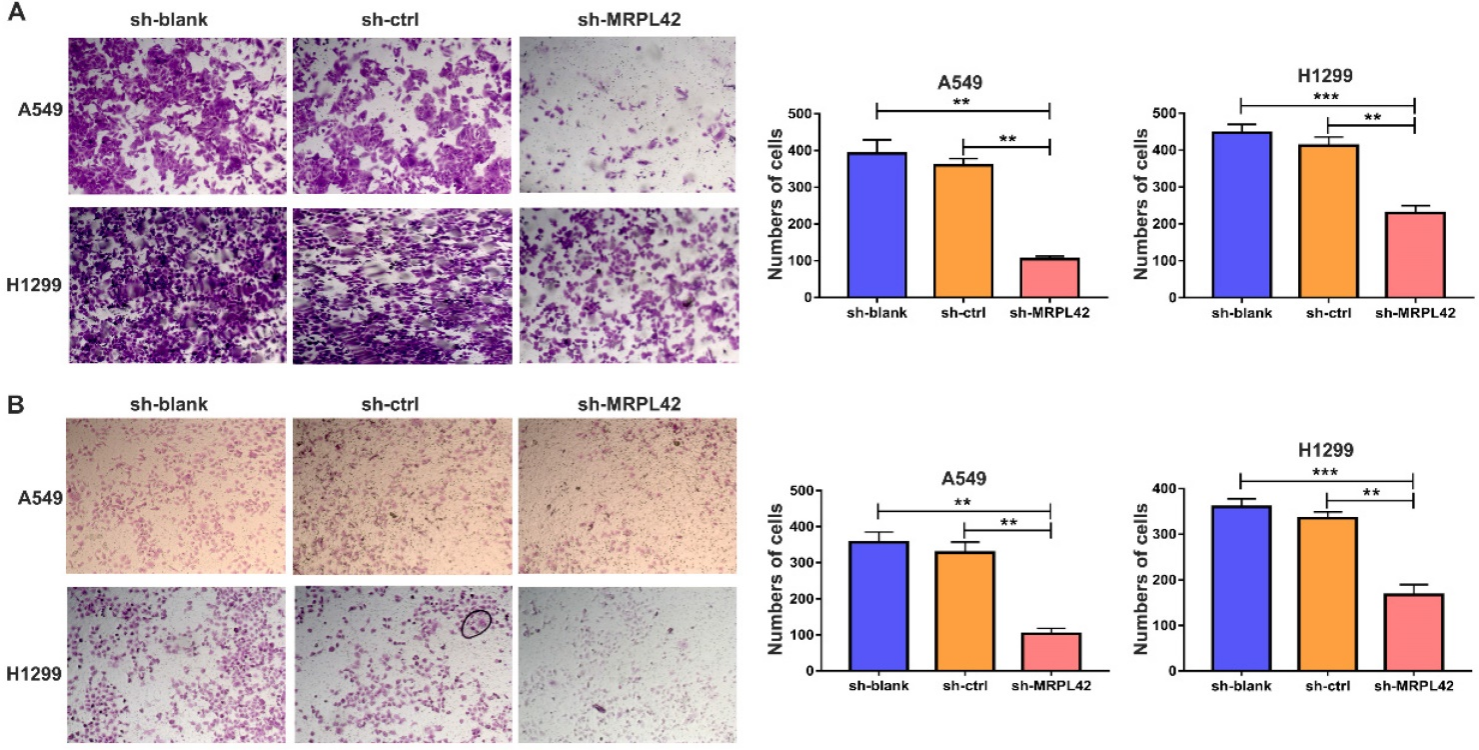
**FAL1 affected migration and metastasis *in vitro*.** (A) Transwell migration assays were conducted with transfected cells and images were acquired at 100× magnification. (B) Transwell invasion assays were conducted with transfected cells and images were acquired at 100× magnification. Data are represented as mean ± SD (**P<0.01; ***P<0.001).

In addition to cell proliferation, cell migration and invasion also play a pivotal role in the development of LUAD. As shown in Figure 3A, the number of migrated cells in sh-MRPL42 group was significantly less than that in sh-blank and sh-ctrl groups. Matrigel assay showed that MRPL42 knockdown markedly reduced the invasion of cells (Figure 3B). In addition, the expression of Vimentin in sh-MRPL42 group was also significantly lower than that in sh-blank group and sh-ctrl group (Figure S1). These results indicated that MRPL42 could facilitate LUAD cell migration and invasion.

### Inhibition of MRPL42 suppressed cell proliferation *in vivo*

**Figure 4:**
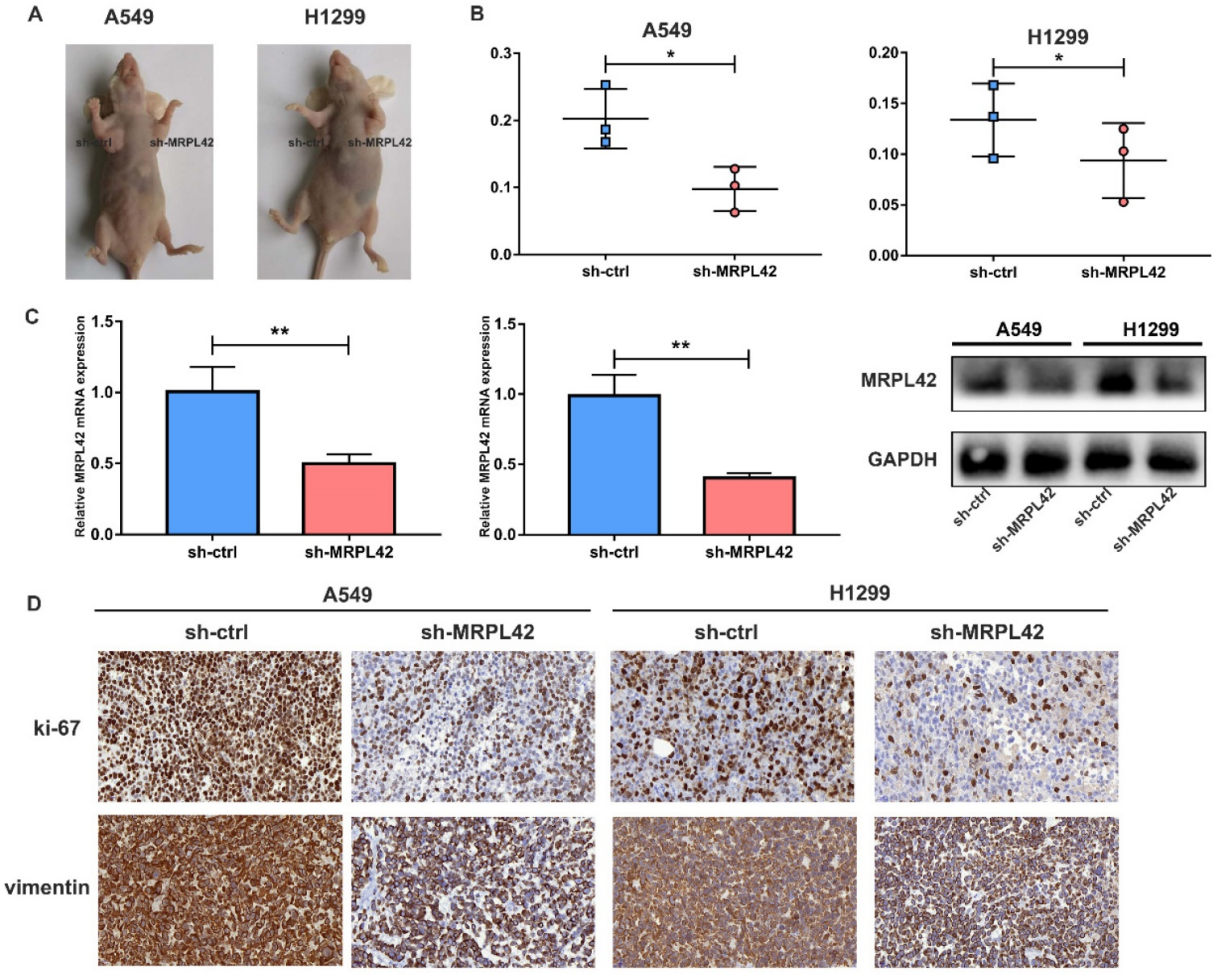
** Downregulation of MRPL42 suppressed xenograft tumorigenicity *in vivo*.** (A) Photo of subcutaneous tumor in nude mice. (B) The weights of tumors were measured and analyzed. (C) The relative expression of MRPL42 mRNA and protein in xenografts was detected by qRT-PCR and Western blot. (D) Immunohistochemical staining against Ki-67and Vimentin was used to determine the effects of MRPL42 in the samples from nude mice. Data are represented as mean ± SD (*P<0.05; **P<0.01).

The transfected cells were inoculated into each nude mouse *in situ* (left, sh-MRPL42 group; right, sh-ctrl group) (Figure 4A). As expected, the tumor weight in sh-MRPL42 group was reduced relative to sh-ctrl group (Figure 4B). In addition, the mRNA and protein levels of MRPL42 in sh-MRPL42 group were significantly reduced (Figure 4C). Results of Ki67 and Vimentin in IHC staining supported that sh-MRPL42 inhibited the proliferation and metastasis of LUAD cells (Figure 4D). These findings indicated that sh-MRPL42 inhibited tumor formation *in vivo*.

### YY1 activated MRPL42 expression in LUAD cells

**Figure 5:**
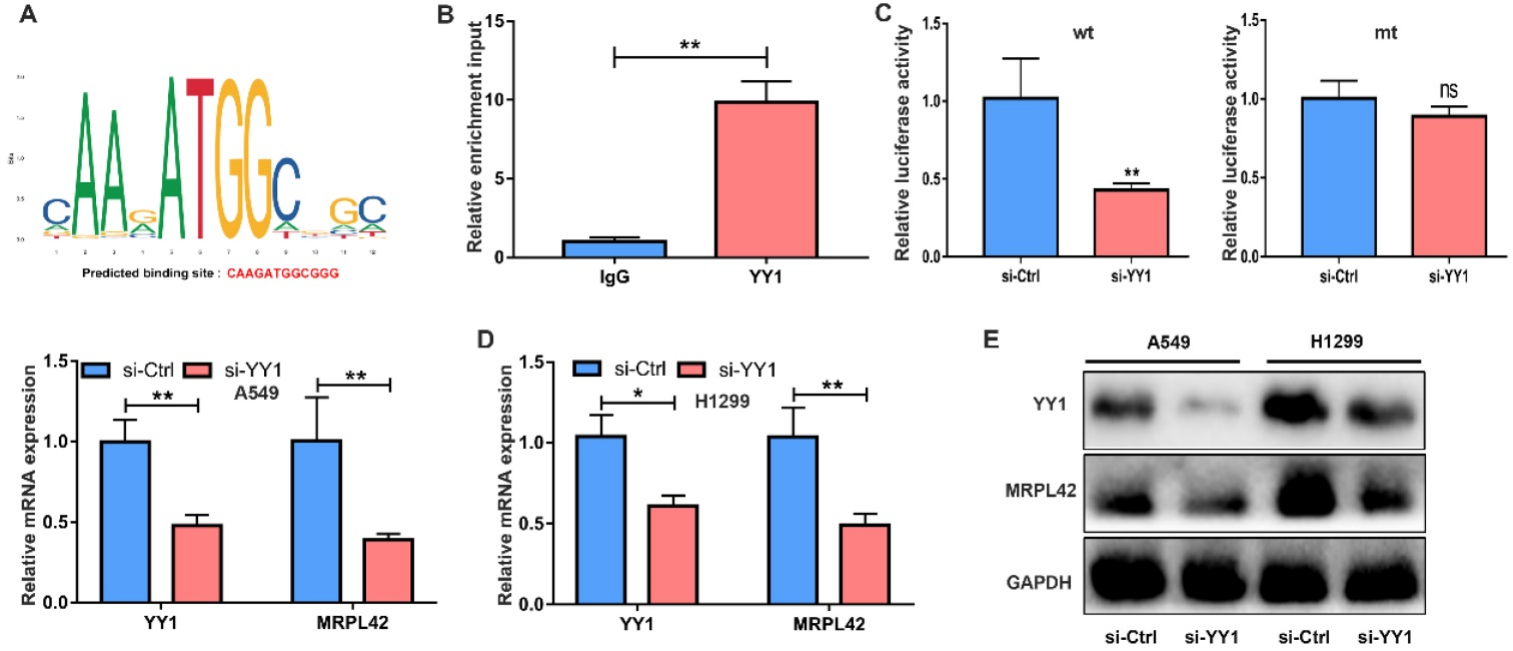
**YY1 was an upstream regulator of MRPL42.** (A) The putative score of YY1 binding to MRPL42 was the highest. Predicted sequence: CAAGATGGCGGG. (B) ChIP assay indicated the YY1 binds to the putative binding site upstream of MRPL42. (C) The interaction between YY1 and MRPL42 promoter was further verified by luciferase reporter assay. (D) The effect of knockdown YY1 by siRNA was identified by qRT-PCR. (E) Levels of MRPL42 mRNA responding downregulated YY1 were detected by qRT-PCR. (E) Levels of YY1 and MRPL42 protein expression after transfection. Data are represented as mean ± SD (*P<0.05; **P<0.01).

To further examine the transcriptional regulation of MRPL42 in LUAD, we searched PROMO database and JASPAR database for potential transcription factors that may regulate MRPL42. A transcription factor YY1 scored higher (Figure 5A). YY1 is a multifunctional transcription factor that can regulate NSCLC cell proliferation and apoptosis 24. ChIP analysis proved that the promoter of MRPL42 was specifically pulled down by YY1 specific antibody, but not by the control antibody (Figure 5B). Dual luciferase reporter assay showed that si-YY1 significantly decreased the fluorescence in wt group (Figure 5C). YY1 was knocked down by siRNA in A549 and H1299 cells, the expression of YY1 and MRPL42 decreased (Figure 5D, 5E and 5F). These findings indicated that YY1 might be a transcriptional activator of MRPL42.

## Discussion

As a malignant tumor with poor prognosis, LUAD is still the main cause of cancer deaths worldwide 25. Although some progress has been made in LUAD, its high recurrence rate and distant metastasis are still the main threats to patient health 26-28. At present, more and more researches focus on discovering new methods for early diagnosis of LUAD and identifying new therapeutic targets.

In this study, we found that the expression of MRPL42 was up-regulated in early-stage (stage I and II) LUAD tissues, and closely related to a poor prognosis. This finding was confirmed again with the clinical data that MRPL42 was closely related to the tumor size and lymph node metastasis in LUAD patients treated at our institution.

Mitochondria provide energy for oxidative phosphorylation, fatty acid oxidation, and synthesis of lipids, nucleotides and amino acids 29. Mammalian mitochondrial ribosomal proteins are encoded by nuclear genes and are responsible for protein synthesis in mitochondria 30. In recent years, studies have revealed the potential roles of multiple mitochondrial proteins in cancer biology. MRPL42, as mitochondrial protein, is located on chromosome 12. MRPL37 is highly expressed in lymphoma tissues and cells, and may be related to cell apoptosis 31. In tumors, MRPL41 could enhance the stability of p53, and in response to growth inhibition conditions, promote p53-induced apoptosis, thereby inhibiting tumor growth and progression 12. MRPL42 downregulation could inhibit glioma cell proliferation by inducing cell cycle arrest and apoptosis 13. Functional studies found that MRPL42 downregulation inhibited LUAD cell growth and metastasis.

Signal transduction pathways such as PI3K/AKT/mTOR and Epithelial-Mesenchymal Transition are highly implicated in cell survival, proliferation and migration. Excessive activation of these pathways is closely related to LC development and metastasis 32, 33. In the present study, after MRPL42 was interfered, the expression of p-Akt and Vimentin in LUAD cells was also inhibited, suggesting that MRPL42 regulates the destiny of LUAD cells.

Through bioinformatics analysis, ChIP and Dual luciferase reporter assay, we found that the transcription factor YY1 regulated the expression of MRPL42. YY1 is a zinc finger transcription factor ubiquitously expressed a series of biological functions, such as embryogenesis, cell proliferation, differentiation and tumorigenesis 34, 35. As a transcription factor, YY1 could activate or inactivate functional gene in tumorigenesis, and participate in the transcriptional regulation of 10% of all mammalian genes 36. YY1 is highly expressed in LC and gears a variety of signaling pathways, such as NF-κB, and PI3K/AKT pathways, to regulate LC progression 24, 37, 38. In addition, YY1 also has the effect of inhibiting tumor progression in LC. For example, YY1 cooperates with AP1 to induce the expression of tumor suppressing molecular chaperone HLJ1, thus reducing cell invasiveness of LUAD cells 39. Therefore, YY1 is either a promoter or a suppressor in tumor development, and this role is decided by downstream factors and tumor types 40. In our study, after knocking down YY1 with siRNA *in vitro*, the mRNA and protein expression of MRPL42 decreased. From this we speculated that YY1 could positively regulate MRPL42, thereby enhancing the cancer-promoting effect of MRPL42.

However, our current research does have some limitations. First, the number of clinical samples was relatively small. Some patients with early-stage LUAD had lesions too small to be sample. Secondly, the patient's blood samples were not collected in time to check the expression of MRPL42 in the plasma.

## Conclusion

YY1 activates MRPL42 expression to promote the proliferation and migration of LUAD cells. This mechanism may lead to the development of new anti-cancer therapies for LUAD.

## Supplementary Material

Supplementary figure and table.

## Figures and Tables

**Table 1:** Correlation between MRPL45 mRNA expression and clinical features in patients with LUAD

Feature	n	MRPL42 mRNA expression		*P* value
		High	Low	
All patients	56	30	26	
**Gender**				0.906
Male	22	12	10	
Female	34	18	16	
**Age (year)**				0.592
≤50	28	14	14	
>50	28	16	12	
**Smoke**				0.489
Yes	21	10	11	
No	35	20	15	
**Tumor size**				
≤3 cm	27	9	18	0.003*
>3 cm	29	21	8	
**Lymph node metastasis**				0.032*
Negative	28	11	17	
Positive	28	19	9	

*P<0.05. MRPL42, mitochondrial ribosomal protein L42; LUAD, lung adenocarcinoma.

## Abbreviations

NSCLC: non-small cell lung cancer
LUAD: lung adenocarcinoma
MRPL42: mitochondrial ribosomal protein L42
YY1: YY1 transcription factor
qRT-PCR: quantitative real-time polymerase chain reaction
shRNA: short hairpin RNA
siRNA: small/short interfering RNA
OD: optical density
