# Supplementary material for: MRPL42 is activated by YY1 to promote lung adenocarcinoma progression
Source: J Cancer. 2021 Mar 1;12(8):2403–11. doi: 10.7150/jca.52277 (PMC7974901; doi:10.7150/jca.52277)
Supplement: Supplementary file 1 — Supplementary figure and table. [file jcav12p2403s1.pdf]

Supplemently figure 1

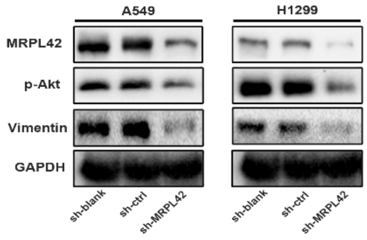

Table 1. Correlation between MRPL45 mRNA expression and clinical features in patients with LUAD

Feature	n	MRPL42 mRNA expression		P value
		High	Low	
All patients	56	30	26	
Gender				0.906
Male	22	12	10	
Female	34	18	16	
Age (year)				0.592
≤50	28	14	14	
>50	28	16	12	
Smoke				0.489
Yes	21	10	11	
No	35	20	15	
Tumor size				
≤3 cm	27	9	18	0.003*
>3 cm	29	21	8	
Lymph node metastasis				0.032*
Negative	28	11	17	
Positive	28	19	9	

*P<0.05. MRPL42, mitochondrial ribosomal protein L42; LUAD, lung adenocarcinoma.
